# Comparison of Micronutrient Deficiencies Following Roux-en-Y Gastric Bypass and One-Anastomosis Gastric Bypass

**DOI:** 10.7759/cureus.71837

**Published:** 2024-10-19

**Authors:** Murad A Khan, Ghulam Siddiq, Muhammad Uneeb, Muhammad Sohaib Khan, Maaz A Yusufi, Najaf N Siddiqi

**Affiliations:** 1 General Surgery, Shifa International Hospital Islamabad, Islamabad, PAK; 2 Surgery, University Hospitals Dorset, Poole, GBR; 3 Colorectal Surgery, University Hospitals Dorset, Poole, GBR

**Keywords:** laparoscopic mini-gastric bypass, malnutrition, micronutrient, micronutrient deficiency, one-anastomosis gastric bypass, roux-en-y gastric bypass (rygb)

## Abstract

Introduction: Obesity is a chronic disease that can be effectively managed with bariatric surgery, such as gastric bypass. Both Roux-en-Y gastric bypass (RYGB) and one-anastomosis gastric bypass (OAGB) surgeries are associated with malnutrition. OAGB is associated with higher weight loss and remission of diabetes. However, it is also associated with a significantly greater incidence of malnutrition than RYGB. We conducted this study to assess the association of both procedures with different micronutrient deficiencies.

Methods: A retrospective single-center cohort study was conducted. Patients undergoing RYGB and OAGB between January 1, 2022, and June 30, 2023, were included. Micronutrient deficiencies following RYGB and OAGB were compared. Vitamin B12, vitamin D, ferritin, and folate levels were assessed preoperatively and between six months to one year after the surgery.

Results: A total of 116 patients underwent gastric bypass surgery, from which 50 (43.1%) patients were excluded due to loss of follow-up, and 66 (56.9%) were included. There were 50 (75.8%) RYGB cases and 16 (24.2%) OAGB cases. There were 24 (36.0%) males. The mean age was 46.2 ± 9.6 years, and the mean preoperative body weight was 131.8 ± 27.9 kg. The mean height was 165.8 ± 9.9 cm, and the mean body mass index (BMI) was 47.7 ± 7.6 kg/m^2^. Both groups were comparable in age, gender distribution, and height. However, patients' mean weight in the OAGB group was higher than in the RYGB group, with a correspondingly higher BMI. Folate deficiency was more common following OAGB (30.8%; N = 4) than RYGB (8.0%; N = 4) (p = 0.028). A fall in folate levels from the initial value was found in the OAGB group, while a rise was found in the RYGB group. However, the two groups had no statistically significant differences in vitamin B12, vitamin D, and ferritin deficiencies (or fall from the initial values).

Conclusion: Both RYGB and OAGB are associated with micronutrient deficiencies. However, OAGB is associated with an increased incidence of micronutrient deficiencies compared to RYGB after six months to one year following the surgery, especially folate deficiency (p = 0.028). Larger, prospective studies and randomized controlled trials are needed, with standardization of the BP limb, the inclusion of multiple ethnicities, and longer follow-up periods.

## Introduction

Obesity was characterized as a chronic disease about four decades ago at the National Institutes of Health consensus conference [1]. Since then, the burden of this disease has reached epidemic proportions, and it continues to increase to this day. It is associated with significant health and economic impact [2]. Obesity is also a component of metabolic syndrome, which is a group of risk factors for cardiovascular disease and diabetes [3]. Optimum management of obesity as a disease, therefore, is of the utmost importance. The conventional treatment of obesity has been lifestyle modifications such as diet and exercise. Certain medications have also been introduced for the management of obesity. However, the most effective therapy for obesity is bariatric surgery, especially when other interventions have failed. Bariatric surgery is associated with greater weight loss than non-surgical treatments and improves comorbidities such as diabetes mellitus [2,4]. The Swedish Obesity Study (SOS) found that bariatric surgery was associated with significantly greater weight loss than conventional treatment. Weight loss at 15 years was 27% with gastric bypass compared to 2% with conventional treatment. Furthermore, bariatric surgery was also associated with a significant reduction in overall mortality [5].

Certain types of bariatric surgery are associated with higher weight loss and beneficial effects on comorbidities than others [4]. However, bariatric surgery is also associated with varying degrees of nutritional and micronutrient deficiencies, depending on the type of surgery [6]. Following bariatric surgery, especially malabsorptive procedures such as gastric bypass, patients can develop micronutrient deficiencies such as vitamin B12, vitamin D, and iron. These necessitate life-long postoperative supplementation to prevent severe deficiencies [6,7].

Mason performed the first gastric bypass in 1966 [8]. Since then, various modifications to the procedure have been described. Laparoscopic Roux-en-Y gastric bypass (RYGB) quickly became the standard bariatric surgery, with robotic bariatric surgery offering further benefits [9,10]. Rutledge introduced the laparoscopic mini-gastric bypass (MGB), also known as the laparoscopic one-anastomosis gastric bypass (OAGB) surgery, in 2001, as a simpler and safer procedure with similar weight loss effects [11]. Both RYGB and OAGB are effective for weight loss and improvement of comorbidities. OAGB is associated with higher weight loss and remission of diabetes; however, it is also associated with a significantly greater incidence of malnutrition than RYGB [12-14]. Most studies comparing RYGB and OAGB have focused on differences in weight loss, improvement of comorbidities, and overall malnutrition [4,12,15]. However, fewer studies have focused on the differences in micronutrient deficiencies following these surgeries [9,16,17]. We conducted a retrospective cohort study to compare specific micronutrient deficiencies following RYGB and OAGB.

This study was previously presented at the 4th Pakistan Society for Metabolic & Bariatric Surgeons (PSMBS) International Conference on April 25, 2024.

## Materials and methods

Study design

A retrospective single-center cohort study was conducted over a period of 18 months between January 1, 2022, and June 30, 2023. Approval was obtained from the Institutional Review Board & Ethics Committee (IRB & EC) at Shifa International Hospitals Ltd. (SIH) (# 147-24). All patients undergoing RYGB or OAGB were included. Chart review was done using electronic medical records. The decision regarding the type of procedure performed had been made on an individualized basis, depending on the patient’s history, as well as their preference. All patients had undergone assessment for nutritional deficiencies both preoperatively and postoperatively, and every patient had micronutrient supplements prescribed following the procedure.

A comparison of postoperative micronutrient deficiencies was done between the two groups. Blood tests for vitamin D, vitamin B12, ferritin, and folate were done at follow-up, between six months and one year after the surgery. Patients were considered to have a deficiency if vitamin B12 < 25 pmol/L, vitamin D < 30 ng/mL, folate < 3.1 ng/mL, and ferritin < 30 ng/mL. These values were selected because they represented the lower limit for each micronutrient at our laboratory. The number of patients with a deficiency of any of these micronutrients in the two groups was then compared. Furthermore, the mean fall in value of these micronutrients from their preoperative value was also calculated among the two groups.

Patients had been advised regarding routine follow-up visits and blood tests. Those patients who did not report for follow-up or did not get the blood tests done during the specified time were called via telephone. Up to three telephone calls on three different occasions were made to attempt to contact these patients. If there was still no response, or the patients did not have the blood tests during the specified time, they were considered lost to follow-up.

Patient selection

All consecutive patients who underwent gastric bypass surgery during the study period at Shifa International Hospital Islamabad were evaluated for inclusion. Patients between the ages of 20 and 80 years were included. Patients undergoing revision bariatric surgery were excluded.

Surgical technique

Roux-en-Y Gastric Bypass

A 4 cm long gastric pouch was fashioned around a 40 Fr bougie. A 100 cm biliopancreatic limb (BP) and a 100 cm alimentary limb (AL) were created. The gastrojejunostomy was kept 3 cm wide, and the jejunojejunostomy was kept 6 cm wide.

One-Anastomosis Gastric Bypass

A 12-cm-long gastric pouch was fashioned around a 40 Fr bougie. The length of the BP limb was 150-180 cm. The gastrojejunostomy was kept 4-5 cm wide.

Statistical methods

Data were analyzed using IBM SPSS Statistics for Windows, version 25 (released 2017; IBM Corp., Armonk, New York, United States). Descriptive statistics were calculated for continuous and categorical data. Frequencies and percentages were calculated for qualitative variables. Mean and standard deviation were calculated for quantitative variables, and an independent samples t-test was done to compare them among the two groups. Pearson chi-square test was done to compare the frequencies of postoperative micronutrient deficiencies among the two groups, as well as the gender distribution. The differences between the preoperative and postoperative values of micronutrients between the two groups were compared using the independent samples t-test. A p-value less than 0.05 was considered to indicate statistical significance.

## Results

A total of 116 patients underwent primary gastric bypass surgery during the study period. There were 88 (75.9%) RYGB cases and 28 (24.1%) OAGB cases. Altogether, 50 (43.1%) patients were excluded from the study due to loss of follow-up or not having the blood tests within six months to one year following the surgery, and the final number of patients included was 66 (56.9%). There were 50 (75.8%) RYGB cases and 16 (24.2%) OAGB cases.

There were 24 (36.0%) males. The majority of patients were female in both groups, with a total of 42 (64.0%) cases. The mean age was 46.2 ± 9.6 years, and the mean preoperative body weight was 131.8 ± 27.9 kg. The mean height was 165.8 ± 9.9 cm, and the mean body mass index (BMI) was 47.7 ± 7.6 kg/m^2^. Both groups were comparable in terms of age, gender distribution, and height (Table 1). However, the mean weight of patients in the OAGB group was higher than in the RYGB group, with a correspondingly higher BMI.

**Table 1:** Demographic details The data are represented as N: number of patients, %: percentage, M: mean, SD: standard deviation, and range. RYGB: Roux-en-Y gastric bypass; OAGB: one-anastomosis gastric bypass; BMI: body mass index. Pearson chi-square test was used to compare the gender distribution between the two groups. Independent samples t-test was used to compare the age, body weight, height, and BMI between the two groups. A p-value less than 0.05 was considered to indicate statistical significance (p < 0.05).

Parameter	Total	RYGB	OAGB	Statistical test value	P-value
N (%)	66 (100)	50 (75.8)	16 (24.2)	-	-
Age (years) (M ± SD) (range)	46.2 ± 9.6 (29-74)	45.7 ± 9.8 (29-74)	47.6 ± 9.2 (35-66)	t(64) = -0.67	0.509
Male N (%): Female N (%)	24 (36.0): 42 (64.0)	18 (36.0): 32 (64.0)	6 (37.5): 10 (62.5)	χ2(1, N = 66) = 0.01	0.914
Body weight (kg) (M ± SD) (range)	131.8 ± 27.9 (90-229)	127.7 ± 25.2 (90-189)	144.4 ± 32.7 (96-229)	t(64) = -2.15	0.036
Height (cm) (M ± SD) (range)	165.8 ± 9.9 cm (145-192)	165.4 ± 8.8 (148-184)	167.3 ± 13.0 (145-192)	t(64) = -0.66	0.510
BMI (kg/m^2^) (M ± SD) (range)	47.7 ± 7.6 (33.7-64.6)	46.54 ± 7.39 (33.7-64.6)	51.2 ± 7.1 (40.6-64.3)	t(64) = -2.22	0.030

Some patients had all four micronutrients tested; however, others had only some of the four tested (Table 2). Folate deficiency was more common following OAGB than RYGB (p = 0.028). However, the two groups had no statistically significant differences in vitamin B12, vitamin D, and ferritin deficiencies. There were no statistically significant differences in the postoperative fall in any of the four micronutrients among the two groups. However, while there was a mean postoperative fall in folate levels in the OAGB group, there was a mean rise in folate levels in the RYGB group.

**Table 2:** Postoperative micronutrient deficiencies and falls from preoperative values The data are represented as n: number of patients with deficiency, N: total number of patients in the subgroup, %: percentage, M: mean, and SD: standard deviation. RYGB: Roux-en-Y gastric bypass; OAGB: one-anastomosis gastric bypass. Pearson chi-square test was used to compare the frequencies of postoperative micronutrient deficiencies between the two groups. Independent samples t-test was used to compare the mean fall in postoperative values of micronutrients between the two groups. A p-value less than 0.05 was considered to indicate statistical significance (p < 0.05).

Parameter	RYGB	OAGB	Statistical test value	P-value
Vitamin B12 deficiency n/N (%)	5/50 (10.0)	2/15 (13.3)	χ2(1, N = 65) = 0.13	0.715
Postoperative fall in vitamin B12 (pmol/L) (M ± SD)	-50.94 ± 136.92	-2.17 ± 113.47	t(60) = -1.14	0.258
Vitamin D deficiency n/N (%)	33/50 (66.0)	12/15 (80.0)	χ2(2, N = 65) = 4.20	0.123
Postoperative fall in vitamin D (ng/mL) (M ± SD)	-0.99 ± 18.14	-11.36 ± 31.21	t(59) = 1.49	0.142
Folate deficiency n/N (%)	4/50 (8.0)	4/13 (30.8)	χ2(1, N = 63) = 4.83	0.028
Postoperative fall in folate (ng/mL) (M ± SD)	-1.27 ± 4.22	1.11 ± 3.02	t(60) = -1.83	0.072
Ferritin deficiency n/N (%)	19/49 (38.8)	4/12 (33.3)	χ2(1, N = 61) = 0.12	0.727
Postoperative fall in ferritin (ng/mL) (M ± SD)	16.72 ± 71.11	92.36 ± 316.30	t(10.238) = -0.79	0.448

## Discussion

Both RYGB and OAGB are associated with postoperative malnutrition and micronutrient deficiencies. However, while several studies compare postoperative malnutrition following these surgical procedures [14,15], there are relatively fewer studies comparing specific postoperative micronutrient deficiencies.

RYGB is a technically challenging surgical procedure with a steep learning curve. Schauer et al. assessed perioperative outcomes of 150 RYGB cases and concluded that the learning curve required to safely perform the surgery and minimize operative time and technical complications was 100 cases [18]. By contrast, OAGB is a relatively simpler procedure associated with shorter operative times, yet it has similar or even better results, especially in improving comorbidities [15]. It requires only one gastrointestinal anastomosis to be created rather than two anastomoses, which is the case in RYGB [16]. Robert et al. conducted a multicenter randomized controlled trial (RCT) to assess the safety and efficacy of RYGB and OAGB. They followed the cases for two years and concluded that OAGB was not inferior to RYGB in terms of weight loss and metabolic improvement. However, the OAGB patients had more overall serious adverse events as compared to RYGB (p = 0.009), as well as more surgery-associated adverse effects (p=0.042). They also found that OAGB was associated with an increase in gastrointestinal side effects, including diarrhea and steatorrhea, as well as an increase in malnutrition [13]. A meta-analysis by Tourky et al., in which they analyzed 13 studies, concluded that OAGB was associated with greater malnutrition than RYGB (p < 0.0001) [14].

Gentileschi et al. conducted a retrospective study to compare malnutrition associated with the two procedures using the Controlling Nutritional Status (CONUT) score [15]. They found that while both procedures were associated with malnutrition, there was no significant difference in the degree of malnutrition associated with RYGB and OAGB. However, they did not assess deficiencies associated with micronutrients [15]. By contrast, we focused on the micronutrient deficiencies associated with both these procedures and found significant differences, specifically in folate levels following OAGB. A recent meta-analysis by Magouliotis et al. found a higher degree of malnutrition associated with OAGB than RYGB [12]. However, they also only looked at overall malnutrition and did not assess associated micronutrient deficiencies following either procedure.

Bhandari et al. performed a retrospective study comparing RYGB and OAGB cases over five years [16]. They studied the nutritional aspects of both procedures, including micronutrient deficiencies. They found that vitamin B12 deficiency was more common among patients following OAGB than RYGB (p = 0.005). Vitamin D deficiency was also more common in the OAGB patients, occurring almost twice as frequently as in the RYGB patients (p = 0.201) [16]. In our study, there was no significant difference between the two groups in either of these. One possible explanation could be that Bhandari et al. created a longer biliopancreatic (BP) limb of 250 cm in their OAGB cases, whereas in our OAGB cases, the BP limb had a length of 150-180 cm. We found an increased frequency of folate deficiency in the OAGB group; however, their study did not look at folate levels. A similar retrospective study comparing the two procedures over two years was done by Zarshenas et al. [17]. They compared weight loss, malnutrition, and gastrointestinal symptoms following the two surgeries. They reported a higher incidence of malnutrition following OAGB, although the difference was not statistically significant. They found that postoperative vitamin B12 and folate deficiency were more common in the OAGB group. They also found a higher incidence of vitamin D deficiency in the RYB group. This was similar to our findings, except that we found a statistically significant difference in the folate deficiency, which was more common following OAGB. They also found that there was a higher incidence of gastrointestinal symptoms in the OAGB group, including diarrhea, reflux (p = 0.029), bile reflux, steatorrhea (p = 0.035), and food intolerance [17]. This could be one possible cause of the increased malnutrition following this surgery [13,17].

Heinonen et al. performed an RCT involving 121 patients undergoing RYGB or OAGB and assessed outcomes one year after the surgery. They found that both procedures had similar effects in terms of weight loss and improvement of comorbidities, although RYGB was associated with better outcomes related to hypertension improvement. They studied micronutrient deficiencies extensively and found that the main difference between the two groups was vitamin D deficiency, which was more common in the OAGB group at 12 months (p = 0.001). By contrast, we found that vitamin D deficiency was similar in both groups. They also found that folate levels increased after one year in the RYGB group but not in the OAGB group. This was closer to our finding of an increased incidence of folate deficiency in the OAGB group. We also found an increase in folate levels following RYGB and a decrease following OAGB. Robert et al. found increased malnutrition with OAGB (p = 0.0034). They studied several different micronutrients but did not measure folate levels. They showed that vitamin B12, vitamin D, and ferritin were deficient in both groups; however, as in our study, the difference was not statistically significant [13]. Tourky et al. similarly found no statistically significant difference in deficiencies of vitamin B12, vitamin D, or ferritin; however, they also did not evaluate folate levels [14].

The variation in the deficiency of different micronutrients that were studied could be due to differences in their mechanisms of absorption, as well as the differences in preexisting stores of these micronutrients in the body. For instance, folate is absorbed mainly in the proximal small bowel. This is due to the requirement of a low pH microenvironment for its absorption, which is achieved in the duodenum and the jejunum [19]. However, vitamin B12 is mainly absorbed from the distal ileum via a complex mechanism involving three transport proteins: intrinsic factor (released from the stomach), haptocorrin, and transcobalamin [20]. Vitamin D is absorbed from the jejunum and ileum, although it can also be produced in the sun-exposed skin [21]. Ferritin is used to assess iron deficiency. Iron is mainly absorbed from the proximal small bowel by the divalent metal transporter DMT1 [22]. These different mechanisms and absorption sites may explain why patients can develop a deficiency of one micronutrient, but have normal levels of another micronutrient, following either RYGB or OAGB.

One possible explanation for the differences in micronutrient deficiencies in the studies mentioned above could be the variability of the BP limb length in the OAGB cases, which is the length of the bypassed small bowel [16,23]. Mahawar et al. surveyed 118 surgeons who had collectively performed 47,364 OAGB procedures. They concluded that longer lengths of the BP limb, especially more than 250 cm, were more likely to be associated with protein-calorie malnutrition severe enough to require surgical revision [23]. Another factor to consider is that there are inherent differences among South Asians as compared to other ethnicities concerning obesity as a disease, as well as the comorbidities associated with it. South Asians have a greater tendency to develop diabetes at lower BMI [24]. The National Institute for Health and Care Excellence (NICE) updated recommendations for bariatric surgery in July 2023. The NICE guidance recommends referral for bariatric surgery for people of South Asian origin at a lower BMI than for other ethnicities because of their increased predisposition to developing central obesity and increased cardiometabolic risk at lower BMI [25]. It is quite reasonable, then, to anticipate a difference in the effects of bariatric surgery, including malnutrition, among this population.

The main limitations of this study are its retrospective nature, the high degree of loss to follow-up, and the relatively wide range of the follow-up period, from six months to one year. The preoperative weight and BMI were higher in the OAGB group, which could result in confounding. The study's main strength was the inclusion of differences between the preoperative and postoperative values of the micronutrients. This allowed us to account for the effect of preexisting deficiencies and excesses of the micronutrients.

## Conclusions

Both RYGB and OAGB are associated with micronutrient deficiencies. Our study showed that OAGB is associated with an increased frequency of folate deficiency compared to RYGB after six months to one year following the surgery (p = 0.028). We also found an increase in the frequencies of vitamin B12 and vitamin D deficiency in the OAGB group, although these differences were not statistically significant. OAGB, therefore, is associated with an increased frequency of micronutrient deficiencies compared to RYGB. Further research is needed to study this aspect of bariatric surgery, ideally with larger, prospective studies and randomized controlled trials, with standardization of the BP limb, the inclusion of multiple ethnicities, and longer follow-up periods. Another potential area of research could be the effect of different postoperative micronutrient supplementation regimens following OAGB, given its increased association with malabsorption.
